# Micro-Expression Recognition Based on Optical Flow and PCANet+

**DOI:** 10.3390/s22114296

**Published:** 2022-06-05

**Authors:** Shiqi Wang, Suen Guan, Hui Lin, Jianming Huang, Fei Long, Junfeng Yao

**Affiliations:** 1 School of Informatics, Xiamen University, Xiamen 361005, China; wangshiqi@stu.xmu.edu.cn (S.W.); gse2020XMU@foxmail.com (S.G.); 24320182203231@stu.xmu.edu.cn (H.L.); yao0010@xmu.edu.cn (J.Y.); 2 Center for Digital Media Computing and Software Engineering, Xiamen University, Xiamen 361005, China; 24320142202428@stu.xmu.edu.cn

**Keywords:** micro-expression recognition, optical flow, PCANet+, deep learning

## Abstract

Micro-expressions are rapid and subtle facial movements. Different from ordinary facial expressions in our daily life, micro-expressions are very difficult to detect and recognize. In recent years, due to a wide range of potential applications in many domains, micro-expression recognition has aroused extensive attention from computer vision. Because available micro-expression datasets are very small, deep neural network models with a huge number of parameters are prone to over-fitting. In this article, we propose an OF-PCANet+ method for micro-expression recognition, in which we design a spatiotemporal feature learning strategy based on shallow PCANet+ model, and we incorporate optical flow sequence stacking with the PCANet+ network to learn discriminative spatiotemporal features. We conduct comprehensive experiments on publicly available SMIC and CASME2 datasets. The results show that our lightweight model obviously outperforms popular hand-crafted methods and also achieves comparable performances with deep learning based methods, such as 3D-FCNN and ELRCN.

## 1. Introduction

Micro-expressions (MEs) are involuntary facial movements with the characteristics of short duration, low intensity, and occurrence in sparse facial action units [1,2]. It is generally believed that the duration of ME is between 1/25 s and 1/2 s [3]. Micro-expression (ME) recognition is a challenging task; even the recognition accuracy by people with specialized training is below 50% [4,5]. Because MEs can reveal genuine emotions people try to hide [1,6], ME recognition has many potential applications in different fields, such as criminal investigation, commercial negotiation, clinical diagnosis, and so on [7,8]. Due to the characteristics of short duration and subtlety, how to extract discriminatory features from ME video clips is a key problem in the task of ME recognition [9]. In recent years, automatic detection and recognition of MEs has become an active research topic in computer vision [10,11,12].

In 2011, Pfister et al. [13] applied LBP-TOP (local binary pattern with three orthogonal planes) [14] to extract dynamic features of MEs on SMIC [12] dataset, and they proposed a benchmark framework for automatic ME recognition. In 2014, Yan et al. [15] established a new ME dataset called CASME2 and used LBP-TOP for ME recognition. Huang et al. [16] proposed a completed local quantization patterns (CLQP) method, which extends LQP by using the sign-based difference, the magnitude-based difference, and the orientation-based difference, and then converts them into binary codes. Wang et al. [17] proposed LBP with six intersection points (LBP-SIP) to obtain a more compact feature representation. The STLBP-IP [18] method proposed by Huang et al. uses integral projection based on difference image and LBP to extract the spatiotemporal features of MEs. In addition, Zong et al. [19] expanded the effectiveness of the LBP Operator by layered STLBP-IP features and reduced the dimension of features by using the sparse learning method.

Lu et al. [20] proposed a Delaunay-based temporal coding model (DTCM) to represent spatiotemporally important features for MEs. Xu et al. [21] proposed a method called Facial Dynamic Map (FDM) to represent the movement patterns of MEs based on dense optical flow. Liu et al. [22] proposed a ME recognition method called Main Directional Mean Optical flow (MDMO), in which a face image is divided into 36 subregions, and the principal direction optical flow of all regions is connected to obtain a low dimensional feature vector. Liong et al. [23] proposed a method of ME detection and recognition by using optical strain information, which can better represent fine, subtle facial movements.

Considering deep learning methods have achieved good performances in facial expression recognition, recently, researchers have attempted to apply deep learning to the task of ME recognition. In [24], Kim et al. proposed to use convolutional neural network (CNN) to encode the spatial features of MEs at different expression-states, and then transfer the spatial features into a Long Short-Term Memory (LSTM) network to learn spatiotemporal features. Peng et al. [25] proposed a dual time-scale convolutional neural network, in which the different stream structures of the network can be used to adapt to ME clips of different frame rates. Li et al. [26] proposed spotting ME apex frames in the frequency domain and fine-tuning a VGG-Face model with magnified apex frames. In the work of [27], Khor et al. introduced an Enriched Long-term Recurrent Convolutional Network (ELRCN) model for micro-expression recognition, which encodes ME features by combining a deep spatial feature learning module and a temporal learning module. Li et al. [28] presented a 3D flow-based CNN (3D-FCNN) model for micro-expression recognition, which uses optical flow together with raw grayscale frames as input to a 12-layer deep network.

Due to the difficulties of ME elicitation and sample annotation, available datasets for training are very small, which limits the performances of deep neural networks for ME recognition. This article investigates the application of a shallow PCANet+ [29] model for the task of ME recognition. PCANet [30] combines principal component analysis (PCA) with CNN architecture. Despite its simplicity, PCANet has achieved promising results in image classification tasks, such as face recognition. As an extension model, PCANet+ eliminates the problem of complete linearity of PCANet and also alleviates the problem of feature dimension explosion by adding a pooling unit between adjacent layers. In this article, we propose a novel ME recognition method (OF-PCANet+) by incorporating the PCANet+ network and dense optical flow calculation. Considering the subtlety of MEs, we first calculate the optical flow from input ME video clips to enhance the motion information; then, we construct multi-channel images by stacking the optical flow fields of consecutive frames and feed them into a two-layer PCANet+ network to learn more powerful spatiotemporal features. A linear SVM is adopted in the classification of ME video clips. Experimental results on publicly available SMIC [12] and CASME2 [15] datasets demonstrate the effectiveness of the proposed method. The main contributions of this article are summarized as follows:

- We propose a lightweight OF-PCANet+ method for ME recognition, which is computationally simple and which can meanwhile produce promising recognition performance.
- We present a spatiotemporal feature learning strategy for ME recognition. Discriminative spatiotemporal features can be learned automatically by feeding stacked optical flow sequences into the PCANet+ network.

The rest of this article is organized as follows. Section 2 gives a brief introduction to optical flow calculation and the PCANet+ model. Section 3 describes our proposed method in detail. Section 4 presents experimental results and discussions, and the conclusions are given in Section 5.

## 2. Preliminaries

Table 1 shows the convention of variable representation adopted in this article. We express the sequential image data of MEs in two forms: (1) an intensity function $I:R^{3}\to R$, which takes three inputs corresponding to the spatial x,y components and the temporal *t* component, respectively; (2) a three-dimensional matrix $I\in R^{N\times M\times L}$, where N,M,L denote the height, width, and length of image data, respectively.

### 2.1. Optical Flow

Optical flow estimation methods take advantage of two assumptions: the constraint of brightness constancy and small motion. The brightness constancy assumes that the gray level of the moving object remains unchanged, and the small motion assumes that the velocity vector field changes very slowly in a short time interval. We suppose that a pixel I(x,y,t) in a video clip will move by Δx,Δy,Δt to the next frame. According to the constraint of brightness constancy mentioned above, the pixel intensity before and after movement is constant, and we can obtain

(1) I(x,y,t)=I(x+Δx,y+Δy,t+Δt).

Based on the constraint of small motion. The right part of Equation (1) can be expanded by Taylor series, as below:

(2) $$\begin{array}{c} I(x+\Delta x,y+\Delta y,t+\Delta t)=I(x,y,t)\\ +\frac{\partial I}{\partial x}\Delta x+\frac{\partial I}{\partial y}\Delta y+\frac{\partial I}{\partial t}\Delta t+\varepsilon , \end{array}$$

where ε represents the high-order term, which can be ignored. Substitute it into Equation (1), we obtain:

(3) $$\frac{\partial I}{\partial x}\Delta x+\frac{\partial I}{\partial y}\Delta y+\frac{\partial I}{\partial t}\Delta t=0.$$

Let *u* and *v* represent the horizontal and vertical components of optical flow, respectively, as $u=\frac{\Delta x}{\Delta t},v=\frac{\Delta y}{\Delta t}$. Substitute them into Equation (3), and we have

(4) $$I_{x}u+I_{y}v+I_{t}=0,$$

where $I_{x}=\frac{\partial I}{\partial x},I_{y}=\frac{\partial I}{\partial y},I_{t}=\frac{\partial I}{\partial t}$ represent the partial derivatives of pixel intensity to *x*, *y*, and *t*, respectively, and (u,v) is called the optical flow field.

### 2.2. PCANet

For a gray-scale image input $I\in R^{N\times M}$, the PCANet extracts a $k_{1}\times k_{2}$ patch around each pixel. Subtract each patch with its patch mean and then reshape it into a vector with length of $k_{1}k_{2}$; we can obtain NM normalized patch vectors. By concatenating them to construct a matrix, we can obtain a normalized patch matrix of I as $P\in R^{k_{1}k_{2}\times NM}$, where each column denotes a single patch vector. Assume that we have a batch of *B* images; concatenating all patches generated from all of the images in the batch similarly gives the patch matrix as $P\in R^{k_{1}k_{2}\times BNM}$. The PCANet aims to minimize a reconstruction error with respect to each patch, as follows.

(5) $$\begin{array}{c} \min_{V\in R^{k_{1}k_{2}\times L_{1}}}{∥P-VV^{T}P∥}_{2}^{2},s.t.V^{T}V=I_{L_{1}}, \end{array}$$

where $L_{1}$ denotes the number of PCA filters and $I_{L_{1}}$ denotes an identity matrix with size of $L_{1}\times L_{1}$. This equation is actually a classic principal component analysis, whose solution is known as the $L_{1}$ principal eigenvectors of $PP^{T}$. Based on this, the *l*-th PCA filter is derived by reshaping the *l*-th principal eigenvectors of $PP^{T}$ into a $k_{1}\times k_{2}$ matrix $W_{l}$. For one PCANet layer with $L_{1}$ PCA filters, the output of the *i*-th image $I_{i}\in R^{N\times M}$ in the batch will be $O_{i}=\left\{I_{i}*W_{1},I_{i}*W_{2},...,I_{i}*W_{L_{1}}\right\}$, where ∗ denotes the convolution operation. Similarly, extracting patches from $O_{i}$ and concatenating them like before, we obtain the input for the next layer $P^{\prime }\in R^{k_{1}k_{2}\times L_{1}BNM}$.

The PCANet could be constructed into a multi-layer architecture, but due to the problem of feature dimension explosion, it usually has many fewer layers than the normal deep neural networks. Here, we only consider a two-layer PCANet, which is widely used. It should be noted that before the final output, there will be a feature encoding layer with the application of hashing and histogram. Let $O_{k}^{1}=I_{i}*W_{k}^{1}\in R^{N\times M}$ be the output of the convolution operation in the 1st layer, where $W_{k}^{1}$ denotes the *k*-th PCA filter in the 1st layer. Then, a hash map will be generated by the following equation to combine the output of each filter.

(6) $$\begin{array}{c} T_{l}=\sum_{k=1}^{L_{2}}2^{k-1}H\left(O_{l}^{1}*W_{k}^{2}\right), \end{array}$$

where $L_{2}$ denotes the number of PCA filters in the 2nd layer, H(·) is a Heaviside step function, whose value is one for positive entries and zero otherwise. $W_{k}^{2}$ denotes the *k*-th PCA filter in the 2nd layer. Let Hist(·) be the function that outputs the histogram vector of the $2^{L_{2}}$ hash labels in a hash map. The final feature vector is expressed as

(7) $$\begin{array}{c} f_{i}=\left[Hist\left(T_{1}\right),Hist\left(T_{2}\right),\cdots ,Hist\left(T_{L_{1}}\right)\right]. \end{array}$$

### 2.3. PCANet+

Because the PCANet layers are completely linearly connected, the lack of nonlinearity could decrease the feature learning effect. The PCANet+ overcomes this problem by adding a mean pooling layer between two consecutive layers, which also helps reduce the feature dimensions. The PCANet+ also extends the original network to support the input of multi-channel images.

Given a multi-channel image $I\in R^{N\times M\times F_{l-1}}$, where N,M denotes the height and the width, respectively. $F_{l-1}$ denotes the number of channels of the input image, which could also denote the number of the filters of the previous layer. Similar to the PCANet, several three-dimensional patches with size of $k_{l}\times k_{l}\times F_{l-1}$ will be generated, where $k_{l}$ denotes the filter size of the *l*-th layer. Thereafter, all of the patches will be reshaped as $P\in R^{k_{l}^{2}F_{l-1}\times BNM}$, which is used for filter learning. Let $F_{l}$ be the number of PCA filters of the current layer and let $W_{k}^{l}\in R^{k_{l}\times k_{l}\times F_{l-1}}$ be the *k*-th learned filter; the output of this layer is expressed as

(8) $$\begin{array}{c} I^{\prime }=\left[\beta \left(I*W_{1}^{l}\right),\beta \left(I*W_{2}^{l}\right),\cdots ,\beta \left(I*W_{F_{l}}^{l}\right)\right]\in R^{N\times M\times F_{l}}, \end{array}$$

where β(·) denotes the mean pooling.

It should be noted that, for the feature encoding layer, based on the one in the PCANet, the PCANet+ also apply the chunking strategy on both the filter level and the image level. For the computation of the hash map, the $F_{l}$ outputs of the filters are divided into $F_{\lambda }$ subsets; then, the hash map for each subset is computed as

(9) $$\begin{array}{c} T_{t}^{l}=\sum_{f=1}^{F_{\lambda }}2^{f-1}H\left(\beta \left(I*W_{\left(t-1\right)\times F_{\lambda }+f}^{l}\right)\right), \end{array}$$

where $t=\{1,2,\ldots ,\frac{F_{l}}{F_{\lambda }}\}$ is the index of the subset. PCANet+ partitions each $T_{t}^{l}$ into $B_{l}$ nonoverlapping blocks, which is histogrammed into $2^{F_{l}}$ bins. Finally, the output of the feature encoding has a size of $\frac{F_{l}}{F_{\lambda }}B_{l}2^{F_{\lambda }}$.

## 3. Method

**Figure 1:**
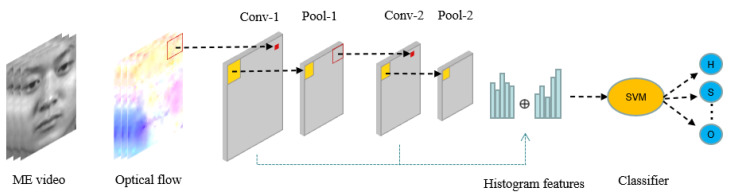
The framework of the proposed ME recognition method.

In this section, we will describe the proposed method for micro-expression recognition in detail. Our method consists of three steps: (1) dense optical flow calculation and multi-channel stacking; (2) feature extraction with PCANet+; (3) classification with support vector machine. Figure 1 shows the overview of our proposed method.

### 3.1. Dense Optical Flow Calculation and Multi-Channel Stacking

The optical flow is a two-dimensional vector field on image plane, which reflects the motion of pixels of two consecutive frames in a video sequence. In order to improve the effect of PCANet+ feature learning, we first perform a dense optical flow calculation on the original cropped ME video clips to enhance the facial motion information.

There are many methods for dense optical flow motion estimation. In this article, we apply the method presented in [31] to dense optical flow calculation, which introduces a subspace trajectory model to keep temporally consistent optical flow. For a single pixel of ME image data $I(x,y,t_{0})$, to compute the sequential optical flow field $u,v\in R^{L-1}$ (*L* denotes the length of ME image sequence), they propose a loss function for optical flow estimation as follows.

(10) $$\begin{array}{c} E\left(x,y,t_{0},u,v\right)=\alpha {\;\int \int \;}_{\Omega }\sum_{t=1}^{L}\left|I\left(x+u\left(t\right),y+v\left(t\right),t\right)-I\left(x,y,t_{0}\right)\right|dxdy\\ +\beta {\;\int \int \;}_{\Omega }\sum_{t=1}^{L}∥\left[u\left(t\right),v\left(t\right)\right]-\sum_{i=1}^{R}q_{i}\left(t\right)\mathrm{lin}\left(u\left(t\right),v\left(t\right)\right){∥}_{2}^{2}dxdy\\ +{\;\int \int \;}_{\Omega }{∥\nabla \mathrm{lin}\left(u\left(t\right),v\left(t\right)\right)∥}_{2}dxdy, \end{array}$$

where $q_{1}\left(t\right),q_{2}\left(t\right),\ldots ,q_{R}\left(t\right):\left\{1,2,\ldots ,L\right\}\to R^{2}$ denote *R* basis trajectories used to construct the trajectory space. $\Omega \in R^{2}$ denotes the image domain. $\mathrm{lin}:R^{2}\to R^{R}$ denotes a map function that maps the optical flow field u(t),v(t) to a new space constructed by the *R* basis trajectories. The first term is the penalty term of the brightness constancy constraint. The second term makes the derived optical flow lie on the basis trajectories. The third term is a total variation-based spatial regularization of the trajectory model coefficients.

**Figure 2:**
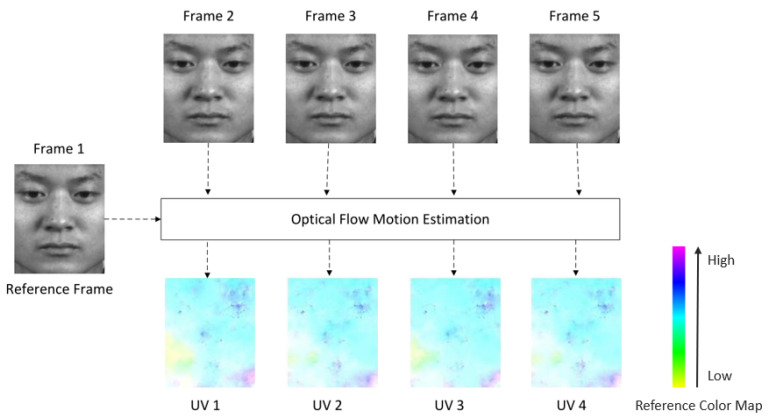
Example of optical flow motion estimation, where we set the first frame of ME image sequence as the reference frame and then compute the optical flow field between the reference frame and the rest of the frames with a subspace trajectory model.

Given an ME image sequence $I\in R^{N\times M\times L}$, we first set its first frame as the reference frame. Based on the optical flow motion estimation method above, we compute the optical flow field sequence of *u* and *v* components as $U,V\in R^{N\times M\times (L-1)}$. Figure 2 shows the results of dense optical flow calculation for a ME video clip (happy class) of CASME2 dataset, in which Frame 1 is the reference frame, and we compute the optical flow field (UV1 to UV4) between the reference frame and the rest of the frames (Frame 2 to Frame 5) by a subspace trajectory model presented in [31]. It should be noted that we use color coding to illustrate the results of optical flow calculation. Different colors indicate different directions, and color saturation indicates the intensity of optical flow. It can be seen that optical flow field can better reflect the movement areas on the face, and it also has a certain effect on filtering the identity information of the face.

**Figure 3:**
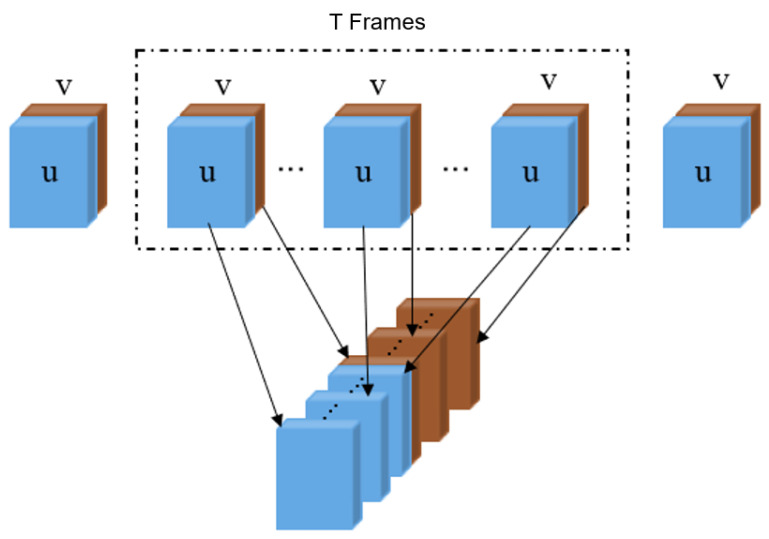
Illustration of stacking optical flow sequences into multi-channel images.

To learn spatiotemporal features by PCANet+ based on optical flow, we conduct a multi-channel stacking operation on the optical flow sequences before they are fed to the PCANet+. Given the computed optical flow sequences $U,V\in R^{N\times M\times (L-1)}$, we use a sliding window with size of *T* and step size of *s* to sample them into several sequence subsets as

(11) $$\begin{array}{c} U=\left\{U_{i}\in R^{N\times M\times T}:U_{i}=U\left(1:N,1:M,\left(i-1\right)s+1:T+\left(i-1\right)s\right),i\in \left[1,\left\lfloor \frac{L-T}{s}\right\rfloor \right]\right\}\\ V=\left\{V_{i}\in R^{N\times M\times T}:V_{i}=V\left(1:N,1:M,\left(i-1\right)s+1:T+\left(i-1\right)s\right),i\in \left[1,\left\lfloor \frac{L-T}{s}\right\rfloor \right]\right\}, \end{array}$$

where $\left|U|=|V|=\lfloor \frac{L-T}{s}\right\rfloor$. Then, each element in U and V will be concatenated to form a stacked input sequence as

(12) $$\begin{array}{c} I=\left\{I_{i}\in R^{N\times M\times (2T)}:I_{i}=U_{i}∥V_{i},i\in \left[1,\left\lfloor \frac{L-T}{s}\right\rfloor \right],U_{i}\in U,V_{i}\in V\right\}, \end{array}$$

where || denotes the matrix concatenating operation through the third dimension. Through the multi-channel stacking operation, the optical flow sequence for each video clip is converted into multi-channel images by stacking adjacent T frames in a sliding window, as shown in Figure 3. These multi-channel images will be fed to PCANet+ network to learn more discriminatory features.

### 3.2. Feature Extraction with PCANet+

PCANet+ can take multi-channel images as input, which therefore makes the capacity of learned filter bank much larger than PCANet [29]. In this article, multi-channel images based on stacking of optical flow sequences are used as input to PCANet+ network for further feature extraction.

For *K* cropped video clips in dataset, after optical flow calculation and stacking process illustrated in Figure 3, we obtain a combined multi-channel image set $I_{\mathrm{all}}=I_{1}\cup I_{2}\cup \ldots \cup I_{K}$, where $I_{i}$ denotes the multi-channel images of the *i*-th video clip. $|I_{\mathrm{all}}|=L_{1}+L_{2}+\ldots +L_{K}$, where $L_{i}$ represents the number of multi-channel images generated from the *i*th video clip after stacking. Here, we set the step size of sliding window as s=(T−1)/2. Then, $I_{\mathrm{all}}$ will be fed to a 2-layer PCANet+ with $D_{1}$ filters (size: $k_{1}\times k_{1}$) in the 1st layer and $D_{2}$ filters (size: $k_{2}\times k_{2}$) in the 2nd layer. To facilitate the succeeding binary hash coding stage in PCANet+, the number of filters $D_{1},D_{2}$ need to be configured to a multiple of $D_{\lambda }$. According to [29], we prefix $D_{\lambda }=8$ in our experiments. Slightly different from the original PCANet+, we apply feature encoding to each PCANet+ layer and concatenate their outputs as the final feature representation, which has $\sum_{l=1}^{2}B_{l}\frac{F_{l}}{F_{\lambda }}2^{F_{\lambda }}$ dimensions in total. Finally, a linear SVM is adopted in the classification of ME video clips.

## 4. Experimental Results and Analysis

To evaluate the proposed method for micro-expression recognition, we conduct comprehensive experiments on two widely used ME datasets, SMIC and CASME2. We first introduce the datasets and evaluation metrics used in experiments, and then we present the experimental results and discussions.

### 4.1. Settings

The SMIC [12] provides three data subsets with different types of recording cameras: SMIC-HS, SMIC-VIS, and SMIC-NIR. SMIC-VIS and SMIC-NIR were recorded by normal speed cameras with 25 fps of visual (VIS) and near infrared (NIR) light range, respectively. Because MEs are rapid facial movements, high speed cameras help to capture more temporal information. In our experiments, the SMIC-HS subset recorded by 100 fps high-speed cameras is used, which contains 164 spontaneous facial ME video clips from 16 subjects. These samples are divided into three ME classes: positive (51 samples), negative (70 samples), and surprise (43 samples).

**Figure 4:**
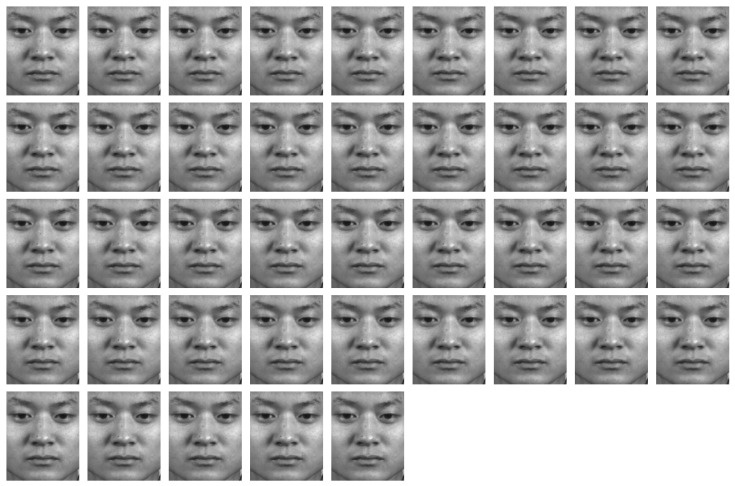
The frames of a sample video clip (happiness) in CASME2 dataset.

The CASME2 [15] dataset consists of 247 spontaneous facial ME video clips with spatial resolution 640 × 480. This dataset was collected by a high-speed camera at 200 fps. As well, MEs of participants were elicited in a well-controlled laboratory environment with four lamps providing steady and high-intensity illumination. The CASME2 dataset includes five ME classes: happiness (32 samples), surprise (25 samples), disgust (64 samples), repression (27 samples), and others (99 samples). The frames of a sample video clip (happiness) in the CASME2 dataset are shown in Figure 4.

The characteristics of two public datasets used in our experiments are summarized in Table 2. To set up a person-independent configuration, leave-one-subject-out (LOSO) cross validation protocol is adopted, where the samples from one subject are used as the testing set, and the samples from the remaining subjects are used as the training set. A linear SVM based on features extracted from PCANet+ is adopted in the classification stage.

Performance metrics such as accuracy, Macro-F1, and Macro-recall, are used in evaluation. Macro-F1 and Macro-recall represent the average F1-score and recall of all classes.

(13) $$\;\mathrm{Accuracy}\;=\frac{\sum_{i=1}^{C}TP_{i}}{\sum_{i=1}^{C}TP_{i}+\sum_{i=1}^{C}FP_{i}}$$

(14) $$P_{i}=\frac{TP_{i}}{TP_{i}+FP_{i}}$$

(15) $$R_{i}=\frac{TP_{i}}{TP_{i}+FN_{i}}$$

(16) $$\;\text{Macro-F1}\;=\frac{1}{C}\sum_{i=1}^{C}\frac{2\times P_{i}\times R_{i}}{\;P_{i}+\;R_{i}}$$

(17) $$\;\text{Macro-recall}\;=\frac{1}{C}\sum_{i=1}^{C}\frac{TP_{i}}{TP_{i}+FP_{i}}$$

where *C* is the class number and $TP_{i}$, $FP_{i}$, and $FN_{i}$ represent true positive, false positive, and false negative of class *i*, respectively.

### 4.2. Effects of Parameters in PCANet+

We need to investigate the hyper-parameters in the OF-PCANet+ method, including the number of frames in stacking (*T*) and the size and number of filters ($\left[k_{1},D_{1}\right]\left[k_{2},D_{2}\right]$). In this article, we build a two-layer PCANet+ model in our method, based on the observation that deeper architectures will not necessarily lead to further performance improvements. In this section, we conduct experiments to examine the influence of these parameters on recognition performance.

#### 4.2.1. The Number of Frames in Stacking

We first examine the number of frames (T) in the process of stacking optical flow sequences. In this experiment, the filter size and number of the network are set to $\left[k_{1},D_{1}\right]=\left[7,32\right]$, $\left[k_{2},D_{2}\right]=\left[9,16\right]$. Table 3 reports the effect of frame stacking number *T* on recognition accuracy.

As shown in Table 3, the performances can be improved by using the operation of frame stacking compared with non-stacking (T=1). The results indicate that multi-frame stacking of optical flow sequences can help the PCANet+ network learn spatiotemporal information, which is very important in ME recognition. When stacking number *T* increases from 1 (i.e., no stacking) to 5, the performances become better, and when *T* increases to 7, the recognition accuracies start to decrease. In the following experiments, we set the best frame stacking number as T=5.

#### 4.2.2. The Size and Number of Filters in Each Layer

**Figure 5:**
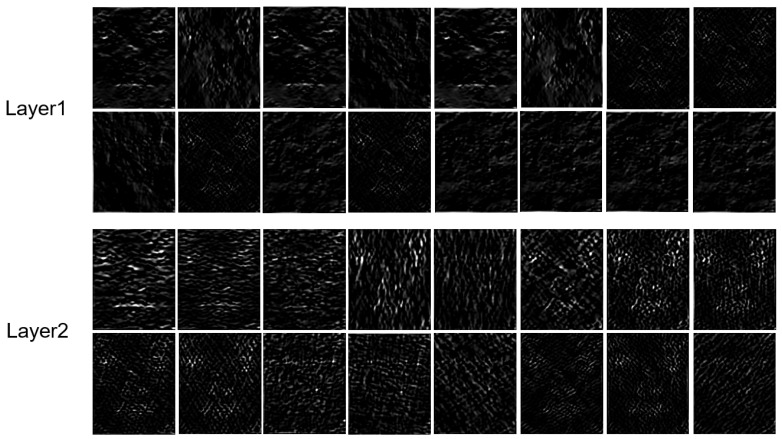
The visualization of feature maps produced in each layer for an input video clip from CASME2 dataset.

We next do experiments to examine the number and size of filters [k,D] used in the OF-PCANet+. The performances in terms of accuracy, macro-F1, and macro-recall with different combinations of [k,D] are reported in Table 4, where k∈{5,7,9,11,13,15} and D∈{8,16,32}. We can see that the proposed method achieves the best recognition performances (in bold) under settings of $\left[k_{1},D_{1}\right]=\left[7,32\right]$, $\left[k_{2},D_{2}\right]=\left[9,16\right]$ on the SMIC dataset and $\left[k_{1},D_{1}\right]=\left[7,16\right]$, $\left[k_{2},D_{2}\right]=\left[7,32\right]$ on the CASME2 dataset. In Table 5, we summarize the best configuration of the PCANet+ network in our method. Figure 5 presents the visualization of feature maps with the parameter of $\left[k_{1},D_{1}\right]=\left[7,16\right]$, $\left[k_{2},D_{2}\right]=\left[9,16\right]$ produced in layer 1 and layer 2, respectively, for an input video clip from the CASME2 dataset. The bright areas have higher motion energy, which means that the facial movements are relatively strong around these areas.

### 4.3. Comparison with Other Methods

To demonstrate the effectiveness of OF-PCANet+, we compare the method with some existing handcrafted methods as well as deep learning methods. The size and number of filters in layer 1 and layer 2 are set to $\left[k_{1},D_{1}\right]=\left[7,32\right]$, $\left[k_{2},D_{2}\right]=\left[9,16\right]$ for SMIC and $\left[k_{1},D_{1}\right]=\left[7,16\right]$, $\left[k_{2},D_{2}\right]=\left[7,32\right]$ for CASME2. Following the experiment settings of [12,15], we re-implement LBP-TOP with 8×8 and 5×5 facial blocks, radius $\left[R_{XY},R_{XT},R_{YT}\right]=\left[4,1,1\right]$. For STLBP-IP, the block size of 4×7 is used for the SMIC dataset, and 8×9 for the CASME2 dataset, as suggested in [18].

Table 6 reports the results of performance comparison of different methods in terms of accuracy, macro-F1, and macro-recall on the SMIC and CASME2 datasets, where N/A indicates that the corresponding performance was not given in the article. We can see that the proposed OF-PCANet+ model outperforms popular hand-crafted methods, i.e., LBP-TOP, STLBP-IP, and KGSL, both on SMIC and CASME2. Furthermore, our method also shows comparable performances with deep learning methods, such as ELRCN [27] and 3D-FCNN [28]. The results indicate that the shallow model of PCANet+ can learn effective spatiotemporal features of micro-expressions based on multi-frame stacking of optical flow sequences.

## 5. Conclusions

In this article, we propose a simple yet effective method OF-PCANet+ for micro-expression recognition by incorporating the dense optical flow calculation with a shallow PCANet+ network. By multi-frame stacking of optical flow sequences as input, discriminative spatiotemporal features can be learned by a two-layer PCANet+ model. Moreover, because the filters can be learned analytically only with the PCA algorithm in each layer, the training process of our method is much simpler than deep learning methods based on back propagation algorithm. The experimental results on SMIC and CASME2 datasets demonstrate the promising performance of the proposed method. In future work, we will try to apply this method to other related tasks, such as behavior recognition and video classification.

## Figures and Tables

**Table 1:** Convention of variable representation.

Variable Symbol	Description
$a\in R^{D}$	A *D*-dimensional real vector.
a(i)∈R	*i*-th element of vector ***a***.
$A\in R^{N\times M}$	A 2-dimensional real matrix with *N* rows and *M* columns.
$A\in R^{N\times M\times L}$	A 3-dimensional real matrix with size of N×M×L.
$A\left(i:j,k:l,n:m\right)\in R^{\left(j-i+1)\times (l-k+1)\times (m-n+1\right)}$	A clipped matrix of $A\in R^{N\times M\times L}$, where i,j,k,l,n,m≥1,i≤j≤N,k≤l≤M,n≤m≤L.
A	A set.
|A|∈N	Size of the set A.

**Table 2:** Detailed information of SMIC and CASME2 dataset.

Dataset	SMIC-HS	CASME2
Subjects	16	26
Sample	164	247
Year	2013	2014
Frame Rate	100	200
Image Resolution	640 × 480	640 × 480
Emotion classes		5 categories:
	3 categories:	happiness (32)
	positive (51)	surprise (25)
	negative (70)	disgust (64)
	surprise (43)	repression (27)
		others (99)

**Table 3:** ME recognition results of OF-PCANet+ with respect to different frame stacking number, *T*.

Frame Stacking Number *T*	SMIC			CASME2		
	Accuracy	Macro-F1	Macro-Recall	Accuracy	Macro-F1	Macro-Recall
1	0.4268	0.3924	0.3890	0.2301	0.2437	0.2316
3	0.6159	0.6184	0.6214	0.4959	0.4960	0.4786
5	0.6280	0.6309	0.6369	0.5203	0.5266	0.5148
7	0.6098	0.6109	0.6131	0.4512	0.4412	0.4270

**Table 4:** ME recognition results of OF-PCANet+ with respect to different number and size of filters [k,D].

$\left[k_{1},D_{1}\right]\left[k_{2},D_{2}\right]$	SMIC			CASME2		
	Accuracy	Macro-F1	Macro-Recall	Accuracy	Macro-F1	Macro-Recall
[5,16][7,16]	0.5854	0.5893	0.5941	0.5000	0.5122	0.4962
[5,32][5,8]	0.5854	0.5880	0.5941	0.5041	0.5047	0.4950
[5,32][5,16]	0.5915	0.5954	0.6036	0.5081	0.5114	0.5020
[5,32][5,32]	0.5793	0.5834	0.5905	0.5163	0.5198	0.5055
[7,16][9,32]	0.6098	0.6127	0.6173	0.5285	0.5272	0.5031
[7,32][5,16]	0.5976	0.6010	0.6084	0.5122	0.5128	0.4950
[7,32][7,16]	0.6098	0.6137	0.6209	0.5041	0.5081	0.4867
[7,32][9,16]	**0.6280**	**0.6309**	**0.6369**	0.5203	0.5266	0.5148
[7,16][7,32]	0.6037	0.6046	0.6096	**0.5325**	**0.5493**	**0.5241**
[7,32][11,16]	0.6037	0.6053	0.6126	0.5285	0.5280	0.5067
[7,32][13,16]	0.5976	0.6007	0.6048	0.4919	0.4931	0.4724
[7,32][15,16]	0.6220	0.6247	0.6310	0.5081	0.5152	0.4931
[9,16][11,16]	0.5915	0.5943	0.6001	0.4268	0.4096	0.4096
[13,16][15,16]	0.6098	0.6131	0.6167	0.4350	0.4250	0.4250

**Table 5:** Summary of the configuration of PCANet+ network.

	Best Configuration For SMIC	Best Configuration For CASME2
$I_{i}$	170×139×10	170×139×10
$W_{1}$	(7×7×10)×32	(7×7×10)×16
$\left(k_{1}\times k_{1}\times 2T\right)\times D_{1}$	Str. 1, Pad. 3	Str. 1, Pad. 3
Pool-1	3×3 Mean Pooling, Str. 1	3×3 Mean Pooling, Str. 1
$W_{2}$	(9×9×32)×16	(7×7×16)×32
$\left(k_{2}\times k_{2}\times 2T\right)\times D_{2}$	Str. 1, Pad. 4	Str. 1, Pad. 3

**Table 6:** Comparisons of different methods.

Method	SMIC			CASME2		
	Accuracy	Macro-F1	Macro-Recall	Accuracy	Macro-F1	Macro-Recall
LBP-TOP [15]	0.4207	0.4266	0.4429	0.4390	0.4297	0.4259
STLBP-IP [18]	0.4329	0.4270	0.4241	0.4173	0.4026	0.4282
KGSL [19]	0.5244	0.4937	0.5162	0.4575	0.4325	0.4437
ELRCN [27]	N/A	N/A	N/A	0.5244	0.5000	0.4396
3D-FCNN [28]	0.5549	N/A	N/A	0.5911	N/A	N/A
OF-PCANet+	0.6280	0.6309	0.6369	0.5325	0.5493	0.5241

## Data Availability

Not applicable.
